# Can We Re-Imagine Research So It Is Timely, Relevant and Responsive? Comment on "Experience of Health Leadership in Partnering with University-Based Researchers in Canada: A Call to ‘Re-Imagine’ Research"

**DOI:** 10.34172/ijhpm.2020.43

**Published:** 2020-03-16

**Authors:** Cecilia Vindrola-Padros

**Affiliations:** Department of Applied Health Research, University College London, London, UK.

**Keywords:** Research Partnerships, Embedded Research, Health System, Rapid Research

## Abstract

Partnerships between academic institutions and healthcare organisations have been proposed as an effective way to integrate academic research findings into changes in health policy and practice. Bowen and colleagues explore these partnerships from a different angle, analysing them in relation to the experiences of health system leaders. The authors made a call to re-imagine research, rethinking how we train applied health researchers, fund health research and evaluation and design studies and collaborations with the health sector. In this paper, I respond to this call by discussing three strategies we can use to make sure our research is timely, relevant and responsive to the needs and context of healthcare organisations: the widespread use of rapid research approaches, the integration of scoping stages in all studies, and the training of applied health researchers to work in the health system and develop collaborative relationships with staff.

## Introduction

A considerable amount of work in the applied health research field currently focuses on finding ways to integrate academic research findings into changes in health policy and practice.^1^ Different models have been tried out around the world, including partnerships between academic institutions and healthcare organisations (with Collaborations for Leadership in Applied Health Research and Care [CLAHRCS] in the United Kingdom, the Veterans’ Health Administration Integrated Health and Research System and Clinical Translational Science Centres in the United States, and the Canadian Institutes for Health Research Health Service Impact Fellowship), the use of knowledge brokers or boundary spanners and embedded research models.^2-7^

One of the goals of these different approaches has been the development of collaborative relationships between academic researchers and the managers and clinicians who will ultimately use the findings in their daily practice.^2-7^ This entails designing different approaches to study co-design and knowledge co-production, where researchers are seen as members of the team and the knowledge of all stakeholders is considered equally valid and useful for informing the studies.^8,9^ Even though there is widespread global use of these models, evaluations are still rare (with the exception of the embedded model and some evaluations of CLAHRCS in the United Kingdom).^10-12^

In their article, Bowen et al^1^ explore these partnerships from a different angle, analysing them in relation to the experiences of health system leaders. According to the authors, these partnerships are perceived as driven by academic researchers, tend to focus on narrow research questions and do not apply a genuine approach to collaboration.^1^ Their study sought to address a gap in knowledge by exploring the experiences of health system leadership with health organisation-university research partnerships.^1^

The authors found a misalignment between healthcare organisations and academic partners, with a conceptualisation of research as unhelpful or irrelevant to decision-making by leaders. Two of the major barriers for the use of research findings were organisational stress and restructuring and the lack of readiness of researchers to work in the fast-paced healthcare environment.^1^ As a result of these findings, the authors made a call to re-imagine research, rethinking how we train applied health researchers, fund health research and evaluation and design studies and collaborations with the health sector. In this paper, I respond to this call by discussing different strategies we can use to make sure our research is timely, relevant and responsive to the needs and context of healthcare organisations (as embedded teams as well as in non-embedded research models).

## The Role of Rapid Research

Timeliness has been highlighted as a factor influencing the utility of research and evaluation findings in healthcare.^13^ In the case of a significant amount of research, only findings shared at time points when they are able to inform decision-making will be able to produce improvements in care.^14-16^ This has prompted the development of a wide range of rapid research approaches characterised by the short duration of research, use of multiple methods for data collection and teams of researchers, formative research designs where findings are fed back while the research is ongoing, and the development of actionable findings to inform changes in policy and/or practice.^17-20^

These might include techniques and approaches such as rapid appraisals, rapid ethnographic assessments, rapid qualitative inquiry, rapid assessment procedures, the rapid assessment, response and evaluation model, and quick, focused or short-term ethnographies.^21,22^ Rapid evaluation methods have also been developed in the form of real time evaluations, rapid feedback evaluations, rapid evaluation methods and rapid-cycle evaluations.^23^

In the United Kingdom, the interest in rapid approaches to research has become more evident, with greater emphasis placed on the need for timely findings and rapid, relevant and responsive research. The National Institute for Health Research has recently funded two rapid service evaluation teams, RSET (Rapid Service Evaluation Team) and BRACE (Birmingham, RAND and Cambridge Evaluation), that aim to reduce the amount of time involved in setting up national service evaluations. I have been involved in the development of an additional center in the United Kingdom called the Rapid Research, Evaluation and Appraisal Lab (RREAL), which seeks to expand and improve the use of rapid research approaches in healthcare (including healthcare systems in high-income countries as well as epidemic response efforts in low- and middle-income countries).

The Center for Medicare and Medicaid Innovation in the United States has created a Rapid Cycle Evaluation Group to test new payment and service delivery models and inform decisions at a policy and practice level in a timely manner.^24^ This shift towards rapid research is mirrored globally by transnational organisations such as the World Health Organization, with their development of methods for rapid evidence synthesis to inform decision-making and the design of rapid advice guidelines for public health emergencies.^25^

These rapid research approaches could be helpful for addressing one of the main barriers identified by Bowen et al^1^ in their article, where health system leaders tended to mention that research was not helpful for improvement efforts because findings were not delivered at a time when they could be used to inform decision-making or researchers were not flexible enough to adapt their studies in connection to changes in the organisations. The rapid approaches mentioned above all seek to address these issues by compressing study timeframes to align their milestones to the changing pace of healthcare organisations, or by building in regular feedback loops so emerging findings can be shared throughout longer studies and inform decisions or changes in practice. These feedback loops are also important for academic researchers as they can be used to obtain feedback from stakeholders in healthcare organisations regarding the relevance of the findings for that particular point in time. As needs and priorities in organisations change, these feedback loops can be used to communicate the need for potential changes in the research to align to these shifts.

## Considering Scoping and Prioritisation as Part of the Research Process

Another way in which we could re-imagine research concerns unpacking what we mean by research. Many researchers maintain traditional concepts of research, where the team designs a protocol, implements data collection, then analysis, and, ultimately, shares the findings through a final report and publications in an academic journal. Our experience in applied health research has pointed to a very important stage that is capable of shaping the entire research process, but has to happen even before the design of the final study protocol (a stage most people would not classify as “research”). We normally call this stage a scoping stage as it allows us to determine, in collaboration with all relevant stakeholders, the scope of the study.

Bowen et al^1^ found that healthcare leaders considered research to be too narrow, not applicable to the local context or answering questions that were important to them. A scoping stage carried out before the design of the study or evaluation allows academic researchers to familiarise themselves with the context of healthcare organisations, their most pressing issues and the main priorities identified by leadership teams. The researchers might carry out some informal data collection in the form of conversations with key stakeholders, observations during meetings or events and documentary analysis. These data can then be used to inform a participatory prioritisation workshop where key stakeholders come together with the research team to discuss, and often prioritise, the areas that need to be included in the study and agree the research questions and study design.

The research team can take the data generated through these discussions and use it to draft the study protocol, which is also reviewed by the group of stakeholders. Our untested assumption is that these discussions and processes of review allow us to develop research that is more relevant to the organisations where we work. Furthermore, as areas are selected jointly and all collaborate in the design of research questions, it gives stakeholders in healthcare organisations, including leaders, a sense of ownership over the study and the findings. This stage requires time and resources, but these are outweighed by the benefits it generates in relation to study design and the building of relationships with staff in healthcare organisations from the beginning of the study.

## Training Researchers

Several academic-healthcare organisation partnerships have tried to address the issue of training. In the United Kingdom, some CLAHRCs have established training programmes or hubs.^26^ Most of this training, however, is aimed at exposing healthcare professionals to research, developing their skills in the use of evidence and evaluation design.^26^ I would argue that in parallel to these training strategies, we need to train academic researchers to be better equipped to work in partnership with healthcare leaders and in changing healthcare landscapes. Bowen et al^1^ found that healthcare leaders visualised academic training as inadequate for preparing researchers for these types of collaborations, lacking ‘soft skills’ and knowledge of the realities of the healthcare system.

Our work on embedded research models pointed to different levels of ‘success’ of embedded researchers depending on their ‘soft skills,’ that is, skills in working collaboratively, engaging staff from different professional groups, and clearly communicating the purpose and findings of research (with limited jargon). These skills can be acquired through practice and over time, but at a system level, we should be doing more to train researchers so they are better equipped to tackle the difficult task of developing and maintaining these collaborative relationships. This is relevant for researchers working as embedded researchers as well as those working in other capacities. The Rapid Research Evaluation and Appraisal Lab at University College London has recently launched a training programme aimed at researchers to develop skills in the design and implementation of rapid research and evaluation, process evaluations, the use of ethnography in healthcare quality improvement and the use of participatory research methods as engagement tools in applied health research.

## Conclusion

A call to re-imagine research has been made and it is up to us to embrace it with the willingness to unpack the concept of research and question our role and preparation as researchers attempting to work in partnership with healthcare organisations. In this brief commentary, I have sought to set out three ways in which we might be able to develop applied health research that is more timely, responsive and relevant to the needs of the healthcare leaders with whom we frequently collaborate. These include the widespread use of rapid research approaches, the integration of scoping stages in all studies, and the training of applied health researchers to work in the health system and develop collaborative relationships with staff. I hope these strategies can represent a useful starting point for those interested in changing healthcare systems through the use of academic research findings.

## Ethical issues

Not applicable.

## Competing interests

Author declares that she has no competing interests.

## Author’s contribution

CVP is the single author of the paper.
